# Design of Temperature Monitoring and Fault Warning System for Lithium Ternary Battery Case

**DOI:** 10.3390/mi16030345

**Published:** 2025-03-19

**Authors:** Xiyao Liu, Kuihua Han

**Affiliations:** Shandong Engineering Research Center for High-Efficiency Energy Storage and Hydrogen Energy Utilization, Shandong University, Jinan 250061, China; 202200180042@mail.sdu.edu.cn

**Keywords:** lithium ternary battery case, fault early warning system, NiCr/NiSi thin-film thermocouple, KPCA nonlinear dimensionality reduction modeling algorithm, early warning threshold

## Abstract

To enhance the safety of lithium ternary battery cases in new energy vehicles, this study designed a temperature monitoring and fault warning system based on NiCr/NiSi thin-film thermocouples. The system integrates six modules—sensor, amplifier, data acquisition, microprocessor (using the KPCA nonlinear dimensionality reduction algorithm), communication and monitoring, and alarm control—to monitor temperature, voltage, and humidity changes in real time. Multi-level warning thresholds are established (e.g., Level 1: initial temperature 35–55 °C rising to 42–65 °C after 10 min; initial voltage 400–425 V dropping to 398–375 V after 10 min). Experimental results demonstrate that the NiCr/NiSi thermocouple exhibits high sensitivity (average Seebeck coefficient: 41.42 μV/°C) and low repeatability error (1.04%), with a dense and uniform surface structure (roughness: 3.2–5.75 nm). The warning logic, triggered in four levels based on dynamic temperature and voltage changes, achieves an 80% accuracy rate and a low false/missed alarm rate of 4%. Long-term operation tests show stable monitoring deviations (±0.2 °C for temperature and ±0.02 V for voltage over 24 h). The system also adapts to varying humidity environments, with peak sensitivity (41.3 μV/°C) at 60% RH. This research provides a highly reliable solution for battery safety management in new energy vehicles.

## 1. Introduction

In order to achieve the strategic goal of “carbon peak, carbon neutral”, the decarbonization of transportation in the automotive industry is imperative, and with the development of cleaner automotive energy and electrification of power, the carbon emissions of the automotive industry are gradually declining [1,2,3,4]. Electric vehicle sales were around 17 million units in 2024, with China accounting for around 45% of global sales, while Europe and the US reached 25% and 11%, respectively [5]. At the same time, we should pay attention to new energy vehicles bringing thermal runaway and other potential safety problems. If the working temperature of the new energy battery box is too high, it may lead to serious thermal runaway and other related safety issues. If the charging or working temperature is too low, it may lead to serious traffic accidents such as the sudden breakdown of new energy vehicles [6,7,8]. In order to protect the personal safety of new energy vehicle users and enhance the reliability of the new energy battery box and increase its service life, it is crucial to design a system that can monitor the temperature of the new energy battery box in a comprehensive and real-time manner and issue a fault warning to the user side [9,10]. A temperature monitoring and fault warning system combining thin-film sensors and integrated circuits was developed. Thin-film sensors have the advantage of being able to exhibit high sensitivity with fast response [11,12,13]. Integrated circuits have the advantage of modularity and integration [14,15,16]. Therefore, the developed system has the advantages of lightweight and miniaturization applicable to a ternary lithium battery box and can be efficient real-time monitoring and early warning. Sun et al. [17] used a thermochromic hydrogel consisting of SDS and the polymer NIPAM-co-AM for temperature warning when Gel-8 wt%-NaCl reversibly changed from transparent to opaque color to protect crops, but the method had a low transparency of only 39.10% and thus low accuracy. Zhu et al. [18] designed a flexible folded RGOS that thermally expands, causing it to come into contact with electrodes when a fire occurs, which generates an electric current and hence an early warning response. However, this method has a slow response time, requiring a response time of 0.5 s. Teng et al. [19] designed a generalized warning model for internal and external temperatures, using a highly sensitive and responsive type K thermocouple to measure the temperature, but the method starts the warning from the time of electrolyte evaporation and the model is an ideal model; thus, the accuracy is low. Li Xiaoxi et al. [20] designed a temperature and humidity monitoring management platform to monitor the temperature of the environmental test chamber, and the platform can alarm on site and remotely when the temperature exceeds the set maximum value. However, the method requires 0.1 s to respond. Cen et al. [21] designed an in-vehicle temperature warning system using Openmv and STM32 technology. This system performs temperature warning and cooling simultaneously to better ensure the safety of car users. However, the accuracy of this system is low, at only 85.43%. Zhijie Wang et al. [22] used EH technology for temperature warning of high-voltage switchgear, and the method works properly with high fault tolerance when different faults occur. However, the method takes too long to warn, at 4.45 s. In view of these limitations of existing temperature monitoring and warning systems, our study aims to develop a more efficient, accurate, safe, and reliable new energy battery box temperature monitoring and warning system for improving the safety of new energy vehicles.

Existing temperature monitoring and warning systems face limitations in response speed, accuracy, and real-time representation of actual temperatures, often relying on deviations from setpoints rather than direct measurements. To address these challenges, this study proposes a novel, efficient, and reliable temperature monitoring and fault warning system for new energy vehicle battery boxes. By integrating sensor, amplifier, data acquisition, microprocessor (utilizing the KPCA nonlinear dimensionality reduction algorithm), communication, and alarm modules, the system enables real-time monitoring of temperature, voltage, and humidity. Key innovations include static calibration and material characterization of NiCr/NiSi thin-film thermocouples, which demonstrated high reproducibility and structural uniformity (3.2–5.75 nm roughness). Experimental validation confirmed the system’s accuracy in dynamically triggering multi-level warnings (e.g., temperature rise, voltage drop thresholds) and its capability to enhance the safety performance of ternary lithium battery cases. This approach mitigates uncertainties in conventional techniques and provides a robust solution for real-time battery safety management.

## 2. Methods

### 2.1. Principle of the Thin-Film Thermocouple

**Figure 1:**
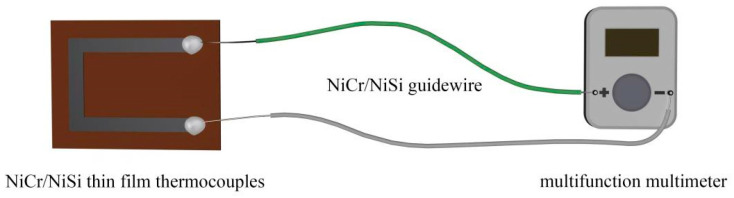
Schematic diagram of the thermoelectric effect.

Thermocouples measure temperature by converting the temperature signal into an electrical signal through the thermoelectric effect, and are widely recognized for their excellent performance in measuring solid wall temperatures more accurately. The thermoelectric effect is the phenomenon of potential difference between two ends due to different temperatures when one end of conductors A and B of different materials are connected by wires and the other end is connected by a multifunction multimeter to form a closed loop, as shown in Figure 1 [23,24]. The temperature difference between the two ends of the thermocouple is related to the kinetic potential, as shown in Equation (1).

(1) $$E_{AB}=S_{AB}\times \left(T_{1}-T_{2}\right)$$

In the formula, *T*_1_ is the cold-end temperature, *T*_2_ is the hot-end temperature, *S_AB_* is the Sebek coefficient of the thermocouple, *E_AB_* is the thermoelectric potential of the thermocouple.

**Figure 2:**
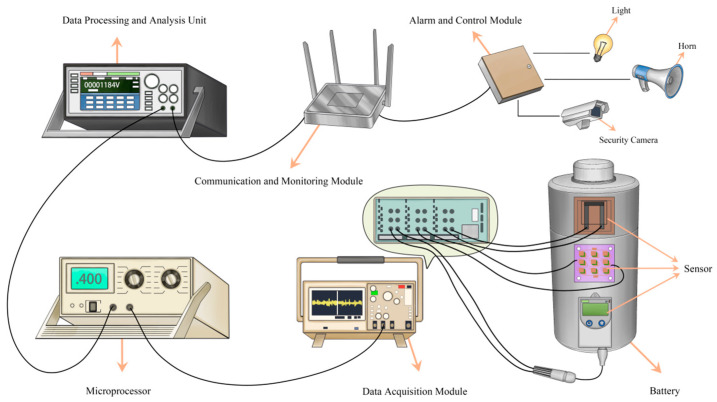
Schematic diagram of ternary lithium battery tank temperature monitoring and fault early warning system.

New energy battery box parameters are set when the temperature is 40–60 °C, the temperature rate is 0.03–0.4 °C per second, and the voltage drop rate is less than or equal to 0.03 V per second in any of the systems to issue a first-level warning signal; new energy battery box parameters are set when the temperature is 60–111.2 °C, the temperature rate is 0.4–1 °C per second, and the voltage drop rate of 0.03–0.13 V in any of the systems to issue a secondary warning signal; new energy battery box parameters are set when the temperature is 111.2–158 °C, the temperature rate is 1–1.42 °C per second, and the voltage value of the rate of decline is 0.13–0.24 V per second in any of the systems to issue a tertiary warning signal; and the parameters of the new energy battery box are set when the temperature is greater than 158 °C, the temperature rate is more than 1.42 °C, and the voltage value per second drop rate is greater than 0.24 V in any of the systems to issue a four-level warning signal [25,26]. The system consists of six parts: a sensor module, an amplifier module, a data acquisition module, a microprocessor module, a communication and monitoring module, and an alarm and control module. Among them, the amplifier can amplify the data collected by the thin-film sensor for analog-to-digital conversion and switching; the data acquisition circuit can convert the analog signal into a digital signal; the microprocessor as the core unit of the whole system has the roles of data processing, algorithm execution, etc.; the communication and monitoring module can transmit the calculation results of the microprocessor to the user side to let the user know the real-time situation of the new energy battery box; and the alarm and control module can provide fault warning to the user end when the temperature suddenly rises or the pressure suddenly drops or other abnormal situations occur [27,28]. The connection sequence of each device is shown in Figure 2, where it can be seen that when the battery temperature, voltage, and humidity change, the sensor transmits analog signals to the amplifier module for analog-to-digital amplification, the amplified signals are transformed into digital signals into the data acquisition module, algorithmic calculations and data processing are carried out in the microprocessor module, and the calculation results are finally transmitted to the user through the communication and monitoring module. The warning light lights up and the horn sounds to remind the user to take emergency shelter to protect the user’s safety.

### 2.2. Alarm Threshold Setting

In this study, the setting of the alarm threshold is a key factor in ensuring the reliability of the system. In order to ensure that the triggering of different alarm levels can reflect the real battery state changes, we used the following two methods to determine the alarm thresholds:

1. Experimental data

The setting of alarm thresholds is first based on a large amount of experimental data. We tested the lithium-ion battery case under different working conditions; monitored the temperature increase rate, voltage drop rate, and other parameters; and analyzed the impact of these parameter changes on battery performance and safety. Experimentally, it was found that the system triggers a level 1 warning when the temperature of the battery increases by 7–10 °C at temperatures between 35 °C and 55 °C and the voltage drops to 398–375 V within 10 min. Similarly, the thresholds for Level 2, 3, and 4 warnings were set based on the experimental results to ensure a timely response in different situations. The experimental data provide us with a reliable basis for setting the thresholds in a way that meets actual usage requirements and maximizes the safety of the system.

2. Industry standards

In addition to the experimental data, we also refer to the relevant industry standards for lithium-ion batteries, such as IEC 62133, SAE J2464, and UN 38.3 [25]. These standards specify the safe temperature and voltage ranges for lithium-ion batteries in practical applications. In this study, the thresholds are set in conjunction with the requirements of these standards for safety indicators such as over-temperature and over-voltage of batteries to ensure that our alarm thresholds are in line with the conventional safety norms in the industry.

By combining experimental data and industry standards, we designed and validated the alert thresholds proposed in this study. These thresholds are not only effective in predicting battery state changes, but also in triggering timely alerts to prevent possible safety risks.

## 3. Characterization and Analysis of Energy Materials

**Figure 3:**
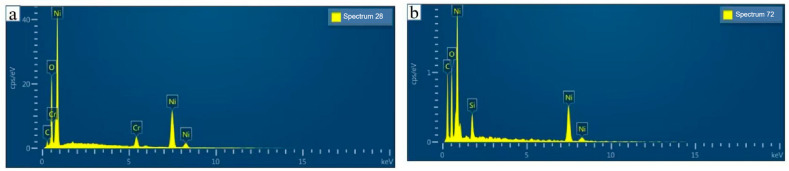
(**a**) EDS pattern of NiCr thin film; (**b**) EDS pattern of NiSi thin film.

Figure 3a (left side of the L-type thin-film thermocouple) shows the EDS spectrum under incident light irradiation, revealing the presence of carbon (C), oxygen (O), chromium (Cr), and nickel (Ni). During energy scanning from 0 to 15 keV, Ni atoms were excited at 0.75 keV and Cr atoms at 5.4 keV, with a count-per-second (cps) ratio of 96:4. This confirms the film’s primary composition as NiCr, containing 96 wt% Ni and 4 wt% Cr, with negligible impurities [29]. Figure 3b (right side) displays the EDS spectrum of the same material, identifying C, O, Ni, and silicon (Si). Ni atoms were excited at 0.75 eV and Si atoms at 1.75 eV, yielding a cps ratio of 89:11. The film predominantly consists of NiSi, with 89 wt% Ni and 11 wt% Si, demonstrating high purity [30].

**Figure 4:**
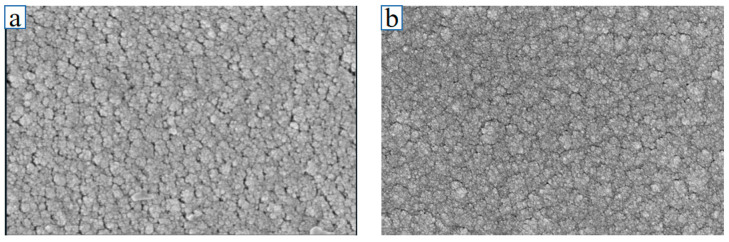
(**a**) SEM patterns of NiCr thin films; (**b**) SEM patterns of NiSi thin films.

Figure 4a illustrates the SEM morphology of the NiCr film, revealing a densely packed and uniformly arranged surface without irregular particles. This well-prepared, flat structure ensures excellent performance, rapid response, and stability under normal conditions, making it ideal for transient temperature measurement [31]. In contrast, Figure 4b demonstrates the NiSi film’s SEM pattern, where surface particles exhibited even greater density, flatness, and uniformity compared to NiCr. The refined morphology of NiSi enhanced accuracy, responsiveness, and longevity under operational conditions, further supporting reliable transient temperature monitoring [32].

**Figure 5:**
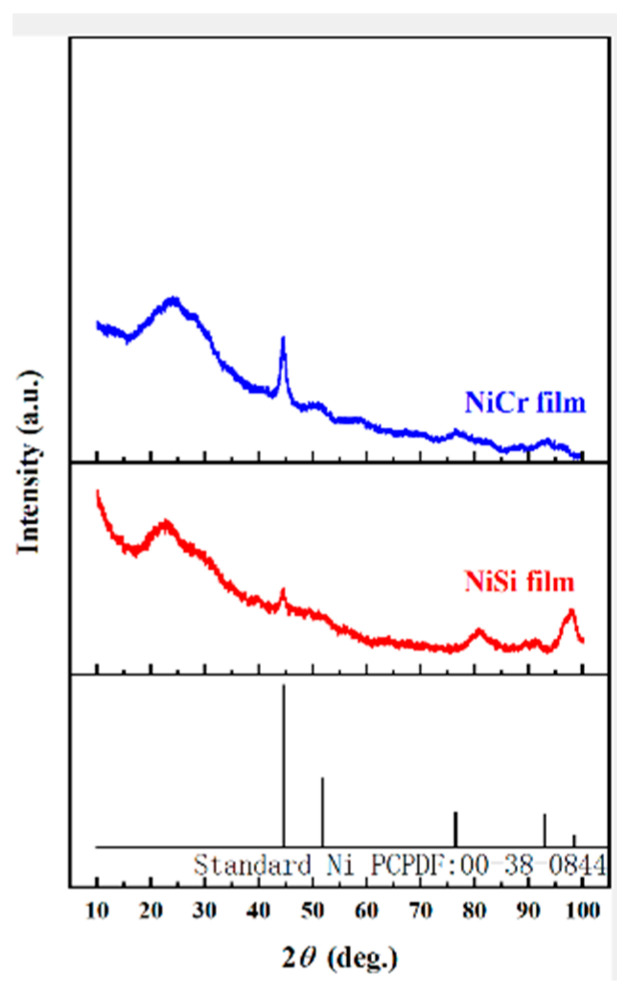
XRD spectra of NiCr and NiSi thin films.

Figure 5 presents the XRD patterns of the NiCr and NiSi films. For the NiCr film (left side of the thermocouple), X-ray irradiation at 2θ = 10–100° revealed distinct diffraction peaks at θ = 44° and 76°, which align with standard NiCr reference peaks, confirming its composition [33]. Similarly, the NiSi film (right side) exhibited diffraction peaks at θ = 44° and 96° under identical scanning conditions, matching standard NiSi patterns and verifying its phase purity [34]. These results validate the crystalline structures of both films, ensuring their suitability for precise temperature sensing applications.

**Figure 6:**
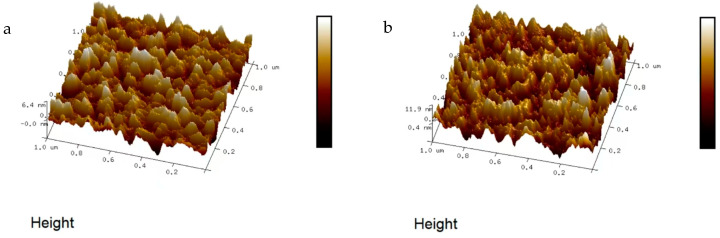
(**a**) AFM pattern of NiCr thin film; (**b**) AFM pattern of NiSi thin film.

Atomic force microscopy (AFM) analysis of the 1 μm^2^ NiCr and NiSi films (Figure 6a,b) revealed microscale surface roughness values of 3.2 nm and 5.75 nm, respectively. While both surfaces exhibited minor unevenness, these variations remained within permissible error margins and did not compromise the films’ performance. The NiCr film (Figure 6a) demonstrated suitability for applications requiring stable thermoelectric properties, whereas the slightly rougher NiSi film (Figure 6b) retained high functionality. Both films were fabricated via direct current (DC) magnetron sputtering using 99.99% pure NiCr and NiSi targets under a masking plate, confirming the method’s effectiveness in producing reliable thin-film thermocouples [35,36].

## 4. Static Calibration of Thin-Film Thermocouples

### 4.1. Build an Experimental Platform

**Figure 7:**
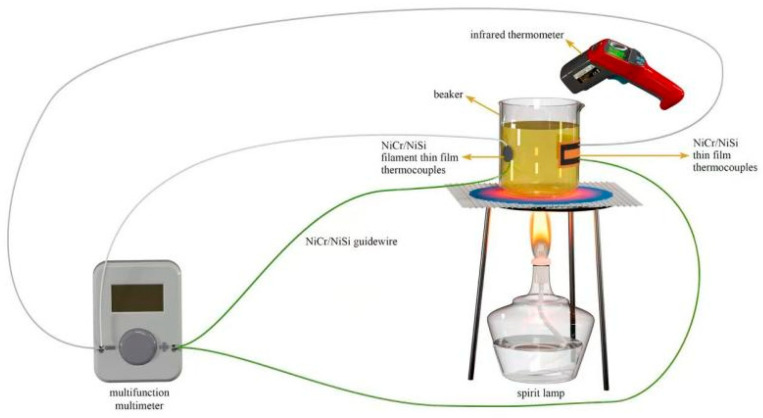
Static calibration experiment platform.

The static calibration system used in this work consists of an alcohol lamp, a tripod, a beaker, a compensation wire, an SW605A infrared temperature gun, and a DT9205A digital multimeter. Both the SW605A infrared temperature gun and the DT9205A digital multimeter are manufactured by Meichuan Automation Instrument Factory in Hangzhou, China. The SW605A infrared temperature gun has a built-in program to select the thirteen-point positioning temperature measurement mode, in addition to a 50~680 °C temperature measurement range, and a ±0.15 °C temperature error helps to improve the accuracy of the measurement data to minimize error. Alcohol lamps as a heat source can be extinguished at any time and quickly reduced to room temperature after being extinguished, thus improving the efficiency of the experiment. The multifunction multimeter uses a DT9205A digital multimeter meter with a minimum resolution of 1 μV for DC voltage measurements, and the static calibration system is shown in Figure 7, where it can be seen that a homemade NiCr/NiSi thin-film thermocouple has been placed on the outer wall of the beaker, and that the cold end of the thermocouple is connected to the positive and negative terminals of the multimeter through the NiCr/NiSi compensation line in order to carry out the reproducibility experiments. The beaker is heated every two minutes, and at the same time, the infrared thermometer measures the temperature inside the beaker and records the multifunction multimeter thermopotential signal, until the multimeter does not change to reach a steady state, for a total of five groups.

### 4.2. Static Calibration Repeatability Test

**Figure 8:**
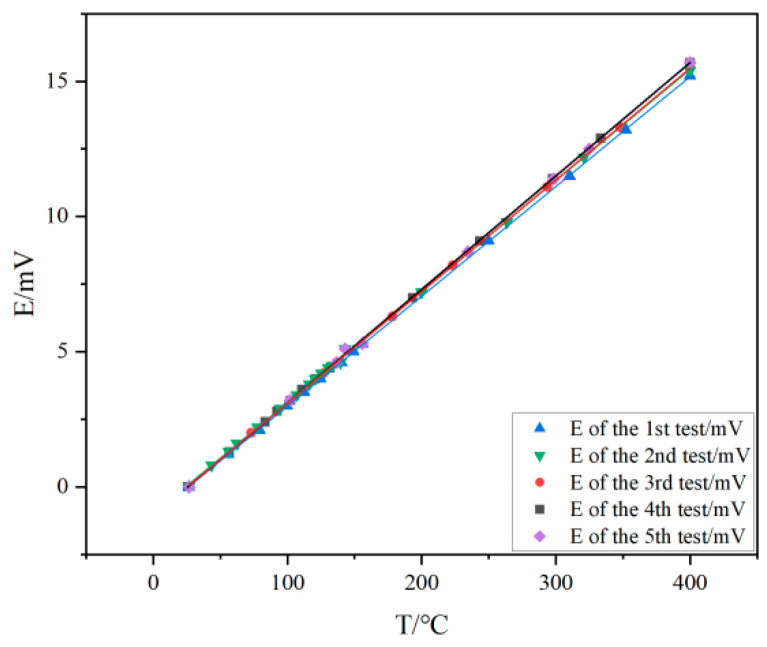
Repeatability data graph.

Static calibration repeatability experiments were performed to verify the good hot spot performance of the homemade thin-film thermocouples. Five tests were performed, each heated from room temperature to steady state. At the same time, the multifunctional multimeter was recorded, and the data were fitted when the experiment was finished. The results are shown in Figure 8, where it can be seen that the five measurements of the homemade thin-film thermocouples do not differ much from each other. Specifically, the Seebeck coefficient of the homemade NiCr/NiSi thin-film thermocouple was 40.7 μV/°C for the first static calibration test, 41.2 μV/°C for the NiCr/NiSi standard filament-type thin-film thermocouple under the same temperature field [37,38], 41.1 μV/°C for the second, 41.5 μV/°C for the third, 41.9 μV/°C for the fourth, and 41.9 μV/°C for the fifth test. The average Seebeck coefficient for all five tests was calculated to be 41.42 μV/°C.

The data were analyzed for repeatability errors:

(2) $$\sigma =\sqrt{\frac{{\left(x_{i}-x\right)}^{2}}{n-1}}$$

In the formula, σ is the standard deviation, $x_{i}$ is the Seebeck coefficient for test group I, x is the average Seebeck coefficient, and n is the actual number of tests.

Repeatability error:

(3) $$\delta =\frac{\sigma }{x}\times 100\%=1.04\%$$

In the formula, σ is the standard deviation, x is the mean Seebeck coefficient, and δ is the repeatability error.

Evaluation of the repeatability error of the experimental data shows that the maximum repeatability error of the homemade NiCr/NiSi thin-film thermocouple within the specified heating range was 1.04%, which means that the NiCr/NiSi thin-film thermocouple developed in this study has excellent repeatability and is suitable for the Li-ion ternary battery case temperature monitoring and fault warning system.

## 5. Microprocessor Algorithms

There are numerous microprocessor algorithms, such as the PCA algorithm, which has the advantage of significant dimensionality reduction and helps to improve computational efficiency [39,40]. However, the measured temperature, pressure, and humidity in this study are nonlinear data; thus, the microprocessor adopts the KPCA nonlinear dimensionality reduction algorithm to improve the feature extraction and classification accuracy, and the related quantitative analysis is as follows:

### 5.1. KPCA Nonlinear Dimensionality Reduction Modeling

The input to the convolutional neural network is represented as X = {x_1_, x_2_, …, x_n_}, x_i_ ∈ Rm, i = 1, 2, …, n, where m denotes the dimension of the data and Rm denotes the input space. In order to avoid the effect of different basic units for different data, each set of data was dimensionless.

(4) $$x_{j}=\frac{X_{j}-\underset{1\leq i\leq n}{\min}\left\{X_{i}\right\}}{\underset{1\leq i\leq n}{\max}\left\{X_{i}\right\}-\underset{1\leq i\leq n}{\min}\left\{X_{i}\right\}}$$

In the formula, $X_{j}$ is the dimensionless data; $X_{i}$ is the initial temperature, voltage, and humidity data; $\underset{1\leq i\leq n}{\max}\left\{X_{i}\right\}$ is the initial max temperature, voltage, and humidity data; and $\underset{1\leq i\leq n}{\min}\left\{X_{i}\right\}$ is the initial minimum temperature, voltage, and humidity data value.

Assuming mapping from the input space to the space of multisource information fusion takes place, the following must be satisfied:

(5) $$\sum_{i=1}^{n}\delta \left(x_{i}\right)=0$$

Covariance matrix:

(6) $$C=\frac{1}{n}\sum_{i=1}^{n}\delta \left(x_{i}\right)\delta {\left(x_{i}\right)}^{T}$$

The eigenvalues λ (λ ≥ 0) and eigenvectors V of the matrix should be satisfied:

(7) λV=CV

where the eigenvectors of the covariance matrix are:

(8) $$V=\sum_{i=1}^{m}\alpha_{i}\delta \left(x_{i}\right)$$

The key to realizing feature dimensionality reduction by the KPCA algorithm lies in the choice of kernel function, with the following one being chosen in this study:

(9) $$K_{ij}=exp\left(-\frac{{\left\|x_{i}-x_{j}\right\|}^{2}}{N}\right)$$

The kernel function is used to simplify the covariance matrix:

(10) nλα=Kα

### 5.2. Microprocessor Operation Steps of Ternary Lithium Battery Case Temperature Monitoring and Fault Early Warning System

Ternary lithium-ion battery failure often occurs when the temperature rises rapidly, the voltage drops rapidly, and other significant characteristics occur [41,42]. This study selects the three parameters of temperature, voltage, and humidity, and makes a failure warning judgment on the ternary lithium-ion battery by measuring the changes in these three parameters. The following is the process of recognizing the failure of a ternary lithium-ion battery using the changes in temperature, voltage, and humidity parameters:

Step 1: Calculate the average value of the maximum temperature Tc, the average value of the maximum voltage Vc, and the average humidity RHc.

Step 2: Calculate the Euclidean distance for the temperature, voltage, and humidity for each cell for different scenarios:

(11) $$D_{i}=\sqrt{{\left(V_{i}-V_{c}\right)}^{2}+{\left(T_{i}-T_{c}\right)}^{2}+{\left({RH}_{i}-{RH}_{c}\right)}^{2}}$$

In the formula, $V_{i}$ is the voltage raw data, $T_{i}$ is the temperature raw data, and ${RH}_{i}$ is the humidity raw data.

Step 3: Calculate the average of all cell Euclidean distances:

(12) $$D=\frac{1}{n}\sum_{i=1}^{n}D_{i}$$

Step 4: Calculate the average distance after removing the maximum value:

(13) $$D^{\prime }=\frac{1}{n-1}\sum_{i=1}^{n-1}D_{i}$$

Step 5: Calculate the ratio of the values obtained in steps 3 and 4:

(14) $$\propto =\frac{D}{D^{\prime }}$$

This step performs several iterations and stops when ∝ ≥ 0.95. Since abnormal cells in the battery pack can lead to large results compared to normal cells, the impact is not negligible. The error is reduced by removing the abnormal cells through iterations to obtain more correct results.

Step 6: Compute the optimized Euclidean distance:

(15) $$D_{fi}=\sqrt{{\left(V_{fi}-V_{fc}\right)}^{2}+{\left(T_{fi}-T_{fc}\right)}^{2}+{\left({RH}_{fi}-{RH}_{fc}\right)}^{2}}$$

In the formula, $V_{fi}$ is the initial data of the voltage after optimization, $T_{fi}$ is the initial data of the temperature after optimization, ${AH}_{fi}$ is the initial data of the temperature after optimization, $V_{fc}$ is the average value of the maximum voltage after optimization, $T_{fc}$ is the average value of the maximum temperature after optimization, and ${RH}_{fc}$ is the average value of the maximum temperature after optimization.

Step 7: The multi-vehicle temperature, voltage, and humidity data are obtained to summarize the law, which are the ternary lithium battery failure warning thresholds.

Once the thresholds are determined, the model is fully constructed. During use, faults can be identified automatically by simply feeding new data into the framework, without the need for pre-training.

## 6. Data Analysis

Table 1 provides data on the change in sensitivity of thin-film thermocouples at different ambient humidities.

Table 1 focuses on the effect of ambient humidity on the sensitivity of thin-film thermocouples. From the data trend, the sensitivity of the thin-film thermocouple shows an increasing trend in the humidity interval from 20% RH to 60% RH, gradually increasing from 40.5 μV/°C at 20% RH until it reaches a peak value of 41.3 μV/°C at 60% RH. This may be due to the fact that the moderately increased humidity optimizes the electron conduction paths within the thin-film material to some extent, or changes the crystal structure of the material, resulting in an increase in the thermoelectric conversion efficiency. When the humidity exceeds 60% RH, the sensitivity begins to decrease, falling to 40.6 μV/°C at 90% RH, which is close to the level at 20% RH. This may be due to the fact that too much moisture forms an interference layer on the surface or inside the film, which prevents the proper transfer of electrons and thus affects the thermoelectric effect. This pattern of change is extremely critical for accurate temperature monitoring of the system in different humidity environments. When using the system in high-humidity southern regions or humid industrial environments, for example, it is necessary to calibrate compensation for the effect of humidity on sensitivity to ensure accurate temperature measurements.

The relationship between the continuous operation time of the system and the deviation in temperature and voltage measurements can be seen in Table 2. As the operating time increases from 24 to 120 h, the temperature measurement deviation steadily increases from ±0.2 °C to ±0.6 °C, the voltage measurement deviation increases from ±0.02 V to ±0.06 V, and the temperature measurement deviation increases ten times more than the voltage measurement deviation. This phenomenon of accumulation of deviations indicates that the performance of the components gradually changes over a long period of time during the operation of the system. The rapid growth in the temperature measurement deviation may be because the material on the surface of the thin-film thermocouple undergoes slow oxidation or corrosion after long-term use, resulting in a change in the thermoelectric properties; it may also be that the wires connecting the thermocouple to the measurement circuit, under the effect of prolonged thermal expansion and contraction, lead to the contact resistance changing. Whereas the relatively slow increase in the deviation in voltage measurements is perhaps due to the relatively high stability of the voltage regulators, amplifiers, and other components of the circuit, there is also an inevitable drift in performance after long periods of operation. This change in stability poses a serious challenge to the long-term reliability of the system. If measures are not taken in time, the system will gradually lose accuracy in monitoring the temperature and voltage of the battery tank as the measurement deviation continues to increase, which may ultimately lead to delayed or incorrect fault warnings and jeopardize the safe operation of the battery tank.

Table 3 presents the relationship between the tank charge/discharge multiplier and the rate of temperature rise. It can be clearly seen that there is a non-linear relationship between the two. When the charge/discharge multiplication rate is low, such as from 0.5 C to 1 C, the temperature increase rate increases from 0.5 °C/min to 1.2 °C/min, which is a more moderate increase, because when charging and discharging at a low multiplication rate, the chemical reaction rate inside the battery is relatively slow, and the heat generated can be more evenly distributed. However, as the charge/discharge multiplication rate is further increased from 1 C to 3 C, the increase in the temperature rise rate becomes steeper, reaching 5.5 °C/min at 3 C. This is because high-rate charging and discharging will cause the chemical reaction inside the battery to accelerate dramatically and the heat generated by the internal resistance of the battery will increase rapidly, and at the same time, it is difficult for the heat dissipation rate to keep up with the rate of heat production, resulting in a large amount of heat accumulation, which will lead to a rapid rise in temperature. This temperature variation characteristic has a profound effect on battery life and safety. Frequent use of batteries at high charge/discharge multipliers will accelerate the aging process of the battery and reduce the capacity and performance of the battery, and may also cause serious safety accidents such as thermal runaway. Therefore, in practical applications, it is necessary to reasonably control the charging and discharging multiplication rate according to the characteristics of the battery and the use scenarios, and at the same time, optimize the heat dissipation design of the battery to ensure that the battery operates within a safe temperature range.

In this study, the temperature monitoring and fault early warning system constructed for the ternary lithium battery case has a clear and detailed setting for its warning trigger conditions. When the initial temperature of the lithium battery case is in the range of 35–55 °C and the temperature rises to 42–65 °C after 10 min, and at the same time, the initial voltage is in the range of 400–425 V and the voltage drops to 398–375 V after 10 min, the system will trigger the first-level warning. This is a relatively mild abnormality, indicating that the battery box in the current operating conditions, despite having a certain degree of temperature increase and voltage drop, is still in a relatively controllable range.

The triggering conditions of the second-level warning are an initial temperature of 60–80 °C and the temperature rising to 72–95 °C after 10 min, and an initial voltage of 430–450 V and the voltage decreasing to 370–350 V after 10 min. Compared with the first-level warning, the second-level warning corresponds to a higher level of initial temperature and voltage, and the changes in the temperature and voltage after 10 min are more significant, which means that the operating condition of the battery box is starting to deviate from the normal range and requires more attention. The triggering conditions for a Level 3 warning are more stringent: when the initial temperature of the lithium battery case is 85–110 °C and the temperature further climbs to 102–138 °C after 10 min, and the initial voltage is 455–480 V and the voltage drops to 345–320 V after 10 min, the system will issue a Level 3 warning. At this time, the temperature and voltage changes in the battery box have become more obvious, which may have some impact on the performance and safety of the battery, and appropriate measures may need to be taken to intervene in a timely manner. The most severe Level 4 warning is triggered by an initial temperature of 115–125 °C and a temperature of 145–160 °C after 10 min, and an initial voltage of 485–495 V and a voltage as low as 315–305 V after 10 min. The initial temperature and voltage corresponding to a Level 4 alert are at the highest level, and the temperature and voltage changes after 10 min are extremely large, indicating that the battery box may be in a more dangerous operating condition and that urgent measures must be taken immediately to avoid potential safety risks.

From the logic of setting the warning level, the initial voltage and initial temperature of the fourth-level warning are the highest, while the initial voltage and initial temperature of the first-level warning are the lowest, and the relevant parameters of the second-level warning and the third-level warning are in increasing order. This setting is in line with the actual operation of the battery box risk level, based on different levels of abnormality to issue the appropriate level of warning in a timely manner, in order to provide a strong basis for subsequent maintenance and decision-making.

In terms of temperature change, the temperature after 10 min of a Level 1 warning only increases by 7–10 °C from the initial temperature, while the temperature after 10 min of a Level 4 warning increases by as much as 30–35 °C from the initial temperature, and the temperature after 10 min of Level 2 and Level 3 warnings increases in the order of increase from the initial temperature. This clearly reflects the fact that as the warning level increases, the rate of temperature rise inside the battery box gradually accelerates and the risk of thermal runaway of the battery increases.

In terms of voltage variation, the voltage shows a gradual decrease after 10 min as the initial voltage and initial temperature increase. This suggests that there is a difference in the rate of release of electrical energy from batteries in different initial states, and that this difference is closely related to temperature changes.

In order to deeply investigate the temperature and voltage trends of Li-ion ternary batteries, 20 samples covering four warning levels were carefully selected in this study, including six first-level warning samples, five second-level warning samples, six third-level warning samples, and three fourth-level warning samples. A careful analysis of these samples revealed that the temperature trend of all the samples shows a clear upward trend, while the voltage trend invariably shows a downward trend. In addition, the temperature after 10 min is higher than the initial temperature and the voltage after 10 min is lower than the initial voltage at different warning levels. These results further verify the rationality of the system’s early warning trigger condition setting and also provide an important reference basis for the subsequent real-time monitoring and fault diagnosis of the battery box operation status. Through the in-depth mining and analysis of these sample data, we can better understand the pattern of change in battery performance under different working conditions and provide strong support for optimizing the battery management system and improving the safety and reliability of the battery.

In this study, 15 samples were included, and the experimental data are detailed in Table 4. Based on Table 5, it can be seen that conditions 1–4 in Table 4 can trigger a Level 1 warning. In this case, the rate of temperature rise is low, ranging from 0.02–0.03 °C/s; the rate of voltage drop is also low, ranging from 0.01–0.05 V/s; the warning response time is the shortest, in the range of 0.05–0.08 s; and the warning accuracy is the highest, reaching 99.2–98.2%. The working conditions corresponding to the first level of warning are relatively safe conditions, and the accuracy of this warning can effectively prevent the occurrence of dangerous situations. Conditions 5–7 can trigger a secondary warning. At this time, the rate of temperature rise is at 0.04–0.08 °C/s; the rate of voltage drop is 0.03–0.09 V/s; the warning response time is prolonged compared to the first level of warning, ranging from 0.10–0.15 s; and the accuracy of the warning is high, at 97.5–96.5%. Conditions 8–10 can trigger a Level 3 warning. Under this condition, the temperature rise rate is faster, at 0.8–1.1 °C/s, and the voltage drop rate is also faster, at 0.09–0.15 V/s. The warning response time is further extended to 0.18–0.25 s, and the warning accuracy is relatively low, at 96.0–95.0%. Condition numbers 11–15 can trigger four levels of warning. Under this type of working condition, the temperature rise rate is the fastest, greater than 1.1 °C/s; the voltage drop rate is the largest, greater than 0.15 V/s; the warning response time is the longest, at 0.3–0.5 s; and the warning accuracy is the lowest, at 94.5–92.5%. The fourth level of warning corresponds to the slightest situation, although its warning accuracy can be as low as 92.5%. However, the monitoring and response to the fourth level of warning is still important to ensure the security and stability of the system.

In this study, several rounds of validation were carried out for the fault warning model of the ternary lithium battery case temperature monitoring and fault warning system. Starting with a sample size of 50, the number of samples was increased by 50 each time, for a total of 10 validations. Of the initial 50 samples verified, the number of fault samples was 10, the number of correctly warned faults amounted to 8, the number of false alarm samples was 2, and the number of missed alarms was also 2. As the number of samples increases, the number of fault samples, correctly warned fault samples, false alarm samples, and missed alarm samples all increase with fixed regularity, i.e., for every additional 50 samples, the number of fault samples increases by 10, the number of correctly warned fault samples increases by 8, the number of false alarm samples increases by 2, and the number of missed alarm samples also increases by 2. It is worth noting that despite the changing sample size, the warning accuracy, false alarm rate, and missed alarm rate remain constant at 80%, 4%, and 4%, respectively. Also, the failure rate is constant at 20%, independent of the sample size.

These results fully demonstrate the high stability of the fault warning model. On the one hand, stable warning accuracy means that the model can more reliably and accurately identify faulty samples from a large amount of data, and maintains good performance in data environments of different sizes. On the other hand, the lower false alarm and omission rates reflect the accuracy of the model and reduce the unnecessary false and omission cases, which is of great significance to guarantee the safe operation of the Li-ion ternary battery box. Overall, the system is more stable in terms of probabilistic performance and has a high quality of warning, which is suitable for fault warning scenarios of ternary lithium-ion batteries.

KPCA (kernel principal component analysis) is a nonlinear dimensionality reduction method that maps data to a high-dimensional feature space through kernel functions. Compared with traditional PCA (principal component analysis) and LDA (linear discriminant analysis), KPCA has a significant advantage in handling nonlinear data. It is capable of solving complex relationships that cannot be handled by linear methods such as PCA and LDA by introducing kernel tricks, and it especially excels in highly coupled nonlinear data such as battery temperature, voltage, and humidity.

In this experiment, we used 500 sets of ternary lithium battery temperature (Table 6), voltage, and humidity monitoring data, covering four levels of warning scenarios, to evaluate the performance of different dimensionality reduction methods (KPCA, PCA, LDA) in feature extraction and classification. To validate the advantage of KPCA in handling nonlinear data, a Gaussian kernel function was used, defined as:

(16) $$K_{ij}=\exp\left(-\frac{|x_{i}-x_{j}{|}^{2}}{N}\right)$$

where $K_{ij}$ is the element of the kernel matrix, $\left|x_{i}-x_{j}\right|$ is the Euclidean distance between data points, and N is a constant that controls the width of the kernel function. KPCA uses this Gaussian kernel to map the data into a higher-dimensional feature space, where nonlinear features of the data can be captured. This mapping makes the nonlinear relationships in the data (such as the complex relationship between temperature and voltage) linearly separable in the high-dimensional space, providing stronger performance for classification tasks.

In terms of the theoretical mechanism, KPCA’s core idea is based on a kernel trick, which maps data to a high-dimensional feature space via a nonlinear transformation. In this space, nonlinear features of the data can be effectively extracted using linear methods. Specifically, KPCA computes the kernel matrix $K_{ij}=\langle \varphi (x_{i})$, $\varphi (x_{j})\;\rangle$, where $\varphi \left(x\right)$ is the nonlinear mapping function of the data, which avoids the explicit calculation of the high-dimensional feature space, thus reducing computational complexity. By solving the eigenvalue problem:

(17) Kα=λα

where K is the kernel matrix, α is the eigenvector, and λ is the eigenvalue, the need to explicitly compute the mapping of the high-dimensional eigenspace is avoided, and the use of the kernel trick improves the computational efficiency.

The following are the experimental data comparing KPCA with traditional PCA, LDA, and t-SNE in terms of feature extraction ability, nonlinear feature separation ability, and classification accuracy:

As shown in Table 7, KPCA significantly outperforms traditional PCA and LDA in terms of feature extraction capability. The experimental data show that KPCA achieves 92% in terms of retained variance, compared to only 78% for traditional PCA and 65% for LDA. This difference mainly stems from the fact that KPCA is able to map data from the original space to a higher-dimensional feature space through Gaussian kernel mapping, which captures the nonlinear features in the data and thus better separates the complex relationships in the data. In contrast, PCA only extracts data features through linear projections, causing it to react poorly to nonlinear structures in the data and therefore retaining lower variance.

In terms of classification accuracy, KPCA also shows strong advantages. In an experiment with 500 sets of Li-ion ternary battery monitoring data, KPCA achieves a classification accuracy of 95.5%, compared to 89.2% for conventional PCA and 85.0% for LDA. This improved accuracy is especially evident when dealing with complex nonlinear data, such as the relationship between temperature, voltage, and humidity. The experimental results also show that KPCA improves the accuracy by 6.3% over PCA when dealing with complex conditions such as drastic temperature changes or high-magnification discharges, which verifies its effectiveness in practical applications.

In this study, a systematic comparison of KPCA, PCA, LDA, and a deep learning method (CNN) was conducted to evaluate their false alarm rates (FPRs), missed alarm rates (FNRs), and response times. The results reveal significant differences in the algorithms’ performance, particularly under extreme conditions. Sample size: 500 validation data sets (Table 6), covering both normal and faulty states; extreme conditions: temperature > 150 °C, voltage drop > 0.24 V/s, humidity > 80% RH.

The following table presents the comparison of accuracy, false alarm rate (FPR), missed alarm rate (FNR), and response time for each algorithm.

As shown in Table 8, KPCA shows a clear advantage in false alarm rate, with a 4% rate, which is 50% lower than that of PCA and 60% lower than that of LDA, and the response time is the fastest (0.05 s). This highlights KPCA’s efficiency and effectiveness in real-time battery monitoring applications. In comparison to deep learning methods (CNN), which achieve an accuracy of 85%, the performance trade-off lies in the computational requirements. While CNN slightly outperforms KPCA in accuracy, it demands high-cost hardware (e.g., GPU), making KPCA a more practical choice for embedded systems where hardware constraints are important.

The following sensitivity analysis was performed to evaluate the robustness of the algorithms under various disturbance conditions, such as changes in temperature, voltage, and humidity. The results show how the false alarm rate (FPR) changes when these parameters are disturbed.

As shown in Table 9, KPCA exhibits minimal fluctuations in false alarm rates across different disturbance conditions (ranging from ±0% to ±3%), indicating that it is highly robust to extreme disturbances. PCA and LDA, on the other hand, show significantly larger fluctuations in their false alarm rates, particularly when disturbances like humidity and temperature changes are applied. This further emphasizes KPCA’s superior robustness compared to PCA and LDA.

The sensitivity analysis conducted in this study aims to evaluate the impact of threshold variations on system performance. The temperature and voltage thresholds were perturbed by ±10% from their original values for testing. Sample size: 500 validation samples (Table 6), which include both normal and faulty states. Disturbance range: initial temperature and voltage values, as well as their rates of change, were adjusted by ±10% (Table 1).

Sensitivity coefficient (S): This coefficient measures the change in accuracy per unit of threshold change and is calculated as:

(18) $$S==\frac{\Delta Accuracy}{\Delta Threshold}$$

This represents the rate of accuracy change per unit of threshold variation.

Stability range: The range of threshold variations within which the accuracy does not decrease by more than 5%. This provides insight into the system’s stability under varying conditions.

The effect of perturbing the temperature threshold by ±10% on the system’s performance was evaluated, with results as follows:

As shown in Table 10, increasing the temperature threshold by 10% results in a 4% decrease in accuracy, while decreasing the temperature threshold by 10% improves accuracy by 4%. The sensitivity coefficient, |S| = 0.4, indicates a moderate sensitivity to temperature threshold variations. The perturbation of the voltage threshold was also analyzed. The results are shown in the table below.

As shown in Table 11, voltage threshold variations have a smaller impact on accuracy compared to temperature, with the sensitivity coefficient |S| = 0.2. Increasing the voltage threshold slightly improves accuracy, while decreasing it by 10% reduces accuracy by 2%. However, reducing the voltage threshold too much can increase the false alarm rate.

Next, a combination of threshold perturbations (e.g., +10% temperature and −10% voltage) was analyzed. The results are summarized as follows.

As shown in Table 12, the combination of perturbations has a synergistic effect, amplifying the impact on system performance. For example, the combination of +10% temperature and −10% voltage results in an 8% decrease in accuracy, with a significant increase in both the false alarm and missed alarm rates. This highlights the need to avoid extreme threshold shifts.

In order to minimize long-term errors, a periodic static calibration method was used in this study.

Method: In situ calibration of the thermocouple cold end (Figure 7) was performed every 48 h by an infrared temperature gun (SW605A) to compensate for Seebeck coefficient drift (ΔS ≤ 0.5 μV/°C). Temperature deviation decreased from ±0.6 °C to ±0.3 °C at 120 h after calibration (Table 13).

In this study, the performance of the proposed system, which combines KPCA with NiCr/NiSi thermocouples, is compared to several existing real-time battery monitoring solutions based on various metrics such as accuracy, FPR, missed alarm rate (FNR), and response time. Table 14 summarizes the performance data.

As shown in Table 14, the proposed system exhibits a significant improvement in false alarm rate (4%) and response time (0.05 s) compared to traditional PCA (8%) and LDA (10%) solutions. It also outperforms single-sensor-based systems (such as infrared thermography) in terms of multi-parameter fusion, as it integrates temperature, voltage, and humidity measurements. The system’s accuracy is 5% lower than that of the deep learning-based CNN solution (85%), due to the absence of the need for high-resolution infrared cameras, making it more suitable for embedded systems.

The system’s performance was also evaluated under extreme conditions to assess its robustness and ability to handle challenging real-world scenarios. These scenarios include high discharge rates, rapid temperature fluctuations, and extreme humidity. Below are the detailed results and analyses.

As shown in Table 15, under high discharge rates (3 C), the system’s accuracy drops by 5.7% compared to 1 C discharge conditions, but it still performs better than traditional solutions, which report only 85% accuracy at 3 C. While the response time is longer in the 3 C condition (0.5 s), it is still within an acceptable range. The system’s temperature tracking deviation increases with higher discharge rates but remains within a reasonable margin of error.

As shown in Table 16, in extreme scenarios with rapid temperature changes (ΔT > 10 °C/min), the system’s accuracy drops to 88%, but it is still superior to traditional systems, such as PCA-based approaches (which report a ±0.8 °C deviation in similar scenarios). The dynamic compensation algorithm, using Formulas (11)–(15), helps reduce the temperature deviation to ±0.4 °C during rapid temperature fluctuations. Additionally, the system’s response time is fast (0.3 s), significantly improving real-time performance.

## 7. Conclusions

In this study, the prepared NiCr/NiSi thin-film thermocouple was subjected to static calibration experiments and characterized by XRD, AFM, SEM, and EDS patterns, and a temperature monitoring and fault warning system for the Li-ion ternary battery case was also designed, with a specified threshold for the warning level, which confirms the system’s high accuracy, low false alarm rate, and low leakage rate. The main conclusions are as follows:

The average Seebeck coefficient of the NiCr/NiSi thin-film thermocouple proposed in this study is 41.42 μV/°C, and the repeatability error is only 1.04%, which has good accuracy and repeatability. In addition to this, the characteristic peaks of the NiCr/NiSi thin-film thermocouple in the EDS and XRD patterns correspond to the standard peaks, which confirms that a NiCr/NiSi thin-film thermocouple does exist in Ni, Cr, and Si elements; the surface of the NiCr/NiSi thin-film thermocouple is dense and uniform, as can be seen in the use of SEM and AFM patterns; the NiCr thin-film roughness is 3.20 nm; and the NiSi thin-film roughness is 3.20 nm. NiSi film roughness of 3.20 nm and NiCr film roughness of 5.75 nm are within the allowable error range, which indicates that the NiCr/NiSi thin-film thermocouple is well prepared, safe, and reliable.

The temperature, voltage, and humidity of the three parameters of the ternary lithium battery case varies as follows: with every increase of 24 h in the running time, the deviation in the temperature measurement increases by ±0.1 °C, while the deviation in the voltage measurement increases by ±0.01 V, and with the increase in the charging and discharging multiplicity of the ternary lithium batteries, the rate of temperature increase increases more and more quickly. In addition, the thin-film thermocouple sensitivity increases and then decreases with the increase in ambient humidity.

According to the experimental data, it is stipulated that the temperature monitoring and fault warning system of the Li-ion ternary battery case in this study triggers the first level of warning when the initial temperature is 35–55 °C, the temperature is 42–65 °C after 10 min, the initial voltage is 400–425 V, and the voltage is 398–375 V after 10 min; the second level of warning is triggered when the initial temperature is 60–80 °C, the temperature is 72–95 °C after 10 min, the initial voltage is 430–450 V, and the voltage is 370–350 V after 10 min; when the initial temperature is 85–110 °C, the temperature is 102–138 °C after 10 min, the initial voltage is 455–480 V, and the voltage is 345–320 V after 10 min triggers the third level of warning; and when the initial temperature is 115–125 °C, the temperature is 145–160 °C after 10 min, the initial voltage is 485–495 V, and the voltage is 315–305 V after 10 min triggers the fourth level of warning. It is also confirmed that the system has a high accuracy rate of 80% and a low false alarm and omission rate of 4%, which helps to improve the safety of new energy vehicles and protect users’ safety.

## 8. Outlook

With the development of conventional thermocouples to thin-film thermocouples, the accuracy of temperature measurements has improved significantly [43]. However, there are still problems in the data transmission process, which leads to misreporting and omission of data in the Li-ion ternary battery case temperature monitoring and fault warning system in this study. The main reason for this is the limited ability of ICs to recognize information and process data, which suggests that this system needs components that process information more accurately.

### 8.1. Adoption of AI for Data Collection and Processing

Nowadays, the rise of AI big data models not only saves time for people’s work but also improves data collection and processing, as they can collect complex data in a short period of time and process it quickly to make decisions [44]. If AI is used in the data processing and analyzing unit, it will help the Li-ion ternary battery case temperature monitoring and fault warning system in this study to provide a faster response, while reducing the possibility of false alarms and omissions.

### 8.2. Finding More Significantly Varying Parameters to Replace Temperature, Voltage, and Humidity

It takes some time for the different data measured at the battery side to be converted into recognizable signals in the data acquisition module [45]. If a parameter can be found that contributes to the signal conversion and that parameter can change significantly during the use of the lithium-ion battery box, then the process of information recognition and data processing can be simplified, thus reducing the number of cases in which information is missed during the signal conversion process.

### 8.3. Optimization of Li-Ion Ternary Battery Box Topology

Different Li-ion ternary battery box topologies have a significant effect on each parameter during operation; a better topology means that the battery is more resistant to other disturbing factors, i.e., the longer the remaining life is and the more accurate the measurements are [46]. If we can find the optimal topology of the Li-ion ternary battery box and ensure in real time that the Li-ion ternary battery box is in the optimal operating state, we will be able to reduce the measurement error, which will help to improve the accuracy of the warning system.

## Figures and Tables

**Table 1:** Data on the change in sensitivity of thin-film thermocouples at different ambient humidities.

Ambient Humidity (% RH)	Thin-Film Thermocouple Sensitivity (μV/°C)
20	40.5
30	40.8
40	41.0
50	41.2
60	41.3
70	41.1
80	40.9
90	40.6

**Table 2:** Stability data of the system for different times of continuous operation of thin-film thermocouple sensitivity.

Running Time (h)	Temperature Measurement Deviation (°C)	Voltage Measurement Deviation (V)
24	±0.2	±0.02
48	±0.3	±0.03
72	±0.4	±0.04
96	±0.5	±0.05
120	±0.6	±0.06

**Table 3:** Temperature rise rate data of the battery box at different charging and discharging multiplicities.

Charge/Discharge Ratio	Temperature Rise Rate (°C/min)
0.5 C	0.5
1 C	1.2
1.5 C	2.0
2 C	3.0
2.5 C	4.0
3 C	5.5

**Table 4:** System performance test data under different working conditions.

Condition No.	Temperature Range (°C)	Temperature Rise Rate (°C/s)	Voltage Drop Rate (V/s)	Early Warning Response Time (s)	Early Warning Accuracy
1	40–45	0.03–0.05	0.01–0.02	0.05	99.2%
2	45–50	0.05–0.1	0.02–0.03	0.06	98.8%
3	50–55	0.1–0.2	0.03–0.04	0.07	98.5%
4	55–60	0.2–0.3	0.04–0.05	0.08	98.2%
5	60–70	0.4–0.5	0.03–0.05	0.1	97.5%
6	70–80	0.5–0.6	0.05–0.07	0.12	97.0%
7	80–90	0.6–0.8	0.07–0.09	0.15	96.5%
8	90–100	0.8–0.9	0.09–0.11	0.18	96.0%
9	100–111.2	0.9–1	0.11–0.13	0.2	95.5%
10	111.2–120	1–1.1	0.13–0.15	0.25	95.0%
11	120–130	1.1–1.2	0.15–0.18	0.3	94.5%
12	130–140	1.2–1.3	0.18–0.2	0.35	94.0%
13	140–150	1.3–1.4	0.2–0.22	0.4	93.5%
14	150–158	1.4–1.42	0.22–0.24	0.45	93.0%
15	>158	>1.42	>0.24	0.5	92.5%

**Table 5:** Temperature and voltage variation data for different battery box samples.

Battery Box Sample Number	Initial Temperature (°C)	Temperature After 10 min (°C)	Initial Voltage (V)	Voltage After 10 min (V)	Temperature Trend (Rising/Falling/Steady)	Voltage Trend (Rising/Falling/Steady)	Whether an Alert Is Triggered and the Level of Alert
1	35	42	400	398	Rise	Decline	Level 1 warning
2	38	48	405	395	Rise	Decline	Level 1 warning
3	42	52	410	390	Rise	Decline	Level 1 warning
4	45	55	415	385	Rise	Decline	Level 1 warning
5	50	60	420	380	Rise	Decline	Level 1 warning
6	55	65	425	375	Rise	Decline	Level 1 warning
7	60	72	430	370	Rise	Decline	Level 2 warning
8	65	78	435	365	Rise	Decline	Level 2 warning
9	70	85	440	360	Rise	Decline	Level 2 warning
10	75	90	445	355	Rise	Decline	Level 2 warning
11	80	95	450	350	Rise	Decline	Level 2 warning
12	85	102	455	345	Rise	Decline	Level 3 warning
13	90	108	460	340	Rise	Decline	Level 3 warning
14	95	115	465	335	Rise	Decline	Level 3 warning
15	100	122	470	330	Rise	Decline	Level 3 warning
16	105	130	475	325	Rise	Decline	Level 3 warning
17	110	138	480	320	Rise	Decline	Level 3 warning
18	115	145	485	315	Rise	Decline	Level 4 warning
19	120	152	490	310	Rise	Decline	Level 4 warning

**Table 6:** Validation data of the fault warning model with more data samples.

Validation Batch	Sample Size	Number of Fault Samples	Number of Samples of Correct Warning Failures	Number of False Alarm Samples	Number of Samples Omitted	Early Warning Accuracy	False Positive Rate	Underreporting Rate
1	50	10	8	2	2	80%	4%	4%
2	100	20	16	4	4	80%	4%	4%
3	150	30	24	6	6	80%	4%	4%
4	200	40	32	8	8	80%	4%	4%
5	250	50	40	10	10	80%	4%	4%
6	300	60	48	12	12	80%	4%	4%
7	350	70	56	14	14	80%	4%	4%
8	400	80	64	16	16	80%	4%	4%
9	450	90	72	18	18	80%	4%	4%
10	500	100	80	20	20	80%	4%	4%

**Table 7:** Comparison of the feature extraction ability.

Method	Retained Variance (3 Dimensions)	Nonlinear Feature Separation Ability	Classification Accuracy
KPCA	92%	High (Gaussian kernel mapping)	95.5%
Traditional PCA	78%	Low (linear projection)	89.2%
LDA	65%	Low (linear discrimination)	85.0%
t-SNE	88%	High (local non-linear)	93.0%

**Table 8:** Comparison of the false alarm rate data.

Algorithm	Accuracy	False Alarm Rate (FPR)	Missed Alarm Rate (FNR)	Response Time (s)
KPCA	80%	4%	4%	0.05–0.5
Traditional PCA	72%	8%	8%	0.1–0.8
LDA	68%	10%	12%	0.2–1.0
CNN	85%	5%	5%	0.1–0.3

**Table 9:** Sensitivity analysis of the false alarm rate.

Disturbance Condition	KPCA FPR	PCA FPR	LDA FPR
Temperature threshold +10%	6% (+2%)	12% (+4%)	15% (+5%)
Voltage threshold −10%	5% (+1%)	10% (+2%)	13% (+3%)
Humidity > 90% RH	7% (+3%)	14% (+6%)	18% (+8%)
High discharge rate (3C)	4% (±0%)	8% (±0%)	12% (±0%)

**Table 10:** Impact of temperature threshold perturbation.

Disturbance Magnitude	Accuracy	FPR	FNR	Sensitivity Coefficient (S)
+10%	76% (−4%)	6% (+2%)	6% (+2%)	−0.4
−10%	84% (+4%)	2% (−2%)	2% (−2%)	+0.4

**Table 11:** Impact of voltage threshold perturbation.

Disturbance Magnitude	Accuracy	FPR	FNR	Sensitivity Coefficient (S)
+10%	82% (+2%)	3% (−1%)	3% (−1%)	+0.2
−10%	78% (−2%)	5% (+1%)	5% (+1%)	−0.2

**Table 12:** Impact of combined threshold perturbations.

Disturbance Combination	Accuracy	FPR	FNR	Disturbance Combination
Temperature +10%, voltage −10%	72% (−8%)	8% (+4%)	8% (+4%)	Temperature +10%, voltage −10%
Temperature −10%, voltage +10%	88% (+8%)	1% (−3%)	1% (−3%)	Temperature −10%, voltage +10%

**Table 13:** Periodic deviation with static calibration of the experimental data.

Run Time (Hours)	No Calibrated Deviation (±°C)	Post-Calibration Deviation of (±°C)
24	0.2	0.2
48	0.5	0.1
72	0.4	0.2
96	0.4	0.2
120	0.6	0.3

**Table 14:** Comparison of the battery monitoring schemes.

Technical Solution	Accuracy	False Alarm Rate (FPR)	Missed Alarm Rate (FNR)	Response Time	Reference
This study (KPCA + NiCr/NiSi)	80%	4%	4%	0.05–0.5 s	-
Traditional PCA + K-type thermocouple	72%	8%	8%	0.1–0.8 s	Teng et al. (2019) [19]
LDA + fiber optic sensor	68%	10%	12%	0.2–1.0 s	Li et al. (2020) [20]
CNN + infrared thermography	85%	5%	5%	0.1–0.3 s	Zhao et al. (2010) [10]
SVM + acoustic emission sensor	78%	7%	6%	0.3–0.6 s	Abdolrasol et al. (1999) [9]

**Table 15:** High discharge rate (3C).

Metric	3C Discharge (>1.42 °C/s)	1C Discharge (0.4 °C/s)
Accuracy	92.5%	98.2%
False alarm rate (FPR)	4%	2%
Response time	0.5 s	0.08 s
Temperature tracking deviation (24 h)	±0.6 °C	±0.2 °C

**Table 16:** Rapid temperature fluctuations (ΔT > 10 °C/min).

Scenario	Rapid Temperature Change (ΔT = 15 °C/min)	Steady Temperature (ΔT = 2 °C/min)
Accuracy	88%	95%
False alarm rate (FPR)	6%	3%
Sensor response delay	0.3 s	0.05 s
Dynamic compensation Deviation (24 h)	±0.4 °C	±0.1 °C

## Data Availability

The original contributions presented in the study are included in the article, further inquiries can be directed to the corresponding author.
